# The effects and mechanism of peiminine-induced apoptosis in human hepatocellular carcinoma HepG2 cells

**DOI:** 10.1371/journal.pone.0201864

**Published:** 2019-01-07

**Authors:** Xu Chao, Guoquan Wang, Yuping Tang, Changhu Dong, Hong Li, Bin Wang, Jieqiong Wu, Jiarong Zhao

**Affiliations:** 1 The Second Affiliated Hospital of Shaanxi University of Chinese Medicine, Xianyang, Shaanxi, P. R.China; 2 The College of Basic Medicine Sciences, Shaanxi University of Chinese Medicine, Xi’an, Shaanxi, P. R.China; 3 The College of Pharmacy, Shaanxi University of Chinese Medicine, Xi’an, Shaanxi, P. R.China; Institute of Biochemistry and Biotechnology, TAIWAN

## Abstract

Peiminine is a compound isolated from *Bolbostemma paniculatum* (Maxim) Franquet (Cucurbitaceae family), which has demonstrated antitumor activities. But its precise molecular mechanism underlying antitumor activity remain elusive. In this study, peiminine-induced apoptosis towards human hepatocellular carcinoma and its molecular mechanism were investigated. MTT assay was employed to assess anticancer effects of peiminine upon Hela, HepG2, SW480 and MCF-7 cell lines. Nuclear staining and flow cytometry were carried out to detect apoptosis induced by peiminine. Mitochondrial membrane potential evaluation and Western blot analysis were performed to investigate the mechanism of peiminine-induced apoptosis. The results showed peiminine reduced the viability of HepG2 cells in a time- and dose-dependent manner and had an IC_50_ of 4.58 μg/mL at 24h. Peiminine significantly increased the percentage of apoptotic cells and the mitochondrial membrane potential dose-dependently in HepG2 cells. The results of Western blotting indicated the expressions of Bcl-2, procaspase-3, procaspase-8, procaspase-9, and PARP decreased in HepG2 cells treated with peiminine, while the expressions of Bax, caspase-3, caspase-8, caspase-9, and cleaved PARP_1_ increased. The result suggests that peiminine can induce apoptosis in human hepatocellular carcinoma HepG2 cells through both extrinsic and intrinsic apoptotic pathways.

## Introduction

Hepatocellular carcinoma (HCC) ranks third among malignancies related to death, and annually occurs in approximately 600,000 individuals worldwide[1]. Although significant advances in frontline cancer research and chemotherapy have been made in treating HCC, many of the proposed drugs cause potent toxic adverse effects[2], thereby significantly hampering their usage in the clinic[3]. Hence, there is an unmet need to identify novel chemical compounds with less adverse effects to combat this devastating disease.

Apoptosis is a type of cell death that is characterized by the preservation of plasma membrane integrity, which prevents local inflammatory reactions and tissue damage[4]. Both intrinsic and extrinsic pathways ultimately converge through the caspase cascade[5–7]. Apoptotic cell death has attracted increasing attention for its role in modulating inhibitory activities of anti-neoplastic compounds[8]. Indeed, an increasing number of reports have demonstrated apoptosis induction as the main mechanism for multiple anticancer agents [9].

Peiminine is a natural compound that is extracted from the bulbs of *Fritillari*a *thunbergii* (Liliaceae family) and *Bolbostemma paniculatum* (Maxim) Franquet (Cucurbitaceae family), and is widely used in traditional Chinese medicine for treating several diseases, including cancer[10]. It has been reported that peiminine repressed colorectal carcinoma tumor growth by inducing apoptosis and autophagy [10,11].

However, the role of peiminine on apoptosis in HCC and its underlying mechanism of action remain largely unknown. The purpose of this study was to elucidate the potential molecular mechanism of apoptosis induced by peiminine.

## Materials and methods

### Chemicals and reagents

Peiminine which purity is 99.8% was purchased from Pure-one Bio Technology, C_O_., LTD., and resolved with injection water. z-DEVD-fmk was purchased from Selleckchem Co., Ltd (Shanghai, China). RPMI-1640 medium, 3-[4,5-Dimethylthiazol-2-yl]-2,5-diphenyltetrazolium bromide (MTT), propidium iodide (PI), 4,6-diamidino-2-phenylindile(DAPI), dimethyl sulfoxide (DMSO), and anti-Bax, Bcl-2, procaspase-3, -8, -9, caspase-3, -8, -9, PARP_1_ (Asp214, 89 kD), PARP_1_ (Asp214, 89 kD) cleaved and β-actin primary antibodies were from Sigma-Aldrich Chemical Co., Ltd (Shanghai, China).

### Cell culture

Hela, HepG2, SW480 and MCF-7 cell lines were purchased from the Cell Bank of Type Culture Collection of Chinese Academy of Sciences (Shanghai, China). Cells were grown in RPMI-1640 medium supplemented with 10% (v/v) heat-inactivated fetal bovine serum (FBS), 100 μg/ml streptomycin and 100U/ml penicillin and maintained at 37°C in a humidified atmosphere containing 5% CO2.

### Cell viability assessment

Cells were seeded in 96-well culture plates at a density of 1×10^4^ cells/well and incubated with peiminine at concentrations of 0, 2, 4, 6, 8, 10, 12, and 14 μg/ml for 24, 48, or 72 h, respectively. 20 μl of MTT solution (5 mg/ml) was added to medium and maintained at 37°C in a humidified atmosphere containing 5% CO_2_ for 4 h. Then the medium was removed and formazan crystals were dissolved in 150 μl DMSO and the absorbance was measured at 570 nm with an *ELx-800* Universal Microplate Reader (BioTek, Winooski, VT). The half-maximal inhibitory concentration (IC_50_) was calculated using SigmaPlot 9.0 software (Systat Software Inc. San Jose, CA). Cell viability (%) was determined as follows:

Cellviability(%)=1−[(ODcontrol−ODtreated)/(ODcontrol−ODblank)]

### Nuclear staining

HepG2 cells treated with peiminine at the concentration of 4 μg/ml for 24h were fixed with paraformaldehyde at 25°C for 10 min, and then incubated with 20μl DAPI at a concentration of 10 μg/ml. Nuclear morphology was analyzed using an Olympus FV1000 fluorescence microscope.

### Detection of DNA fragmentation

3 ml HepG2 cell suspension was plated into 6-well plates (1×10^6^ cells/well) and treated with peiminine at concentrations of 0, 2, 4, 6 μg/ml for 24 h. Then Cells were lysed and genomic DNA was extracted using phenol/chloroform/isoamyl alcohol (25: 24: 1), and RNA was removed by RNaseA. DNA (30 μg) was separated by 1.5% agarose gel electrophoresis and DNA fragmentation patterns were visualized.

### Apoptosis assessment

An annexin V-FITC (fluorescein isothiocyanate) Kit (BD Co., MA, Ltd, USA) was used to quantify the percentage of cells undergoing apoptosis. Briefly, HepG2 cells were treated with peiminine at various concentrations for 24 h. Then cells were collected, washed twice with 400 ml of binding buffer, and incubated in 100 ml reagent mix containing 1 ml annexin V-FITC conjugate and 10 ml PI in the dark for 15 min at 25°C. The samples were subjected to flow cytometer (BD Bioscience, MA, USA) using the CellQuest software (BDIS) to quantify the percentage of cells at different stages.

### Cell cycle detection

3 ml HepG2 cell suspension plated into 6-well plates (1×10^6^ cells/well) were treated with peiminine at concentrations of 0, 2, 4, 6 μg/ml and incubated in humidified incubator at 37°C with 5% CO_2_ for 24 h. Then the cells were harvested and fixed with 70% ethanol overnight, and incubated with 1 ml PI solution (20 μg/ ml in PBS with 1% Triton X-100) containing RNaseA at 37°C away from light for 30 min. Cell cycles were assessed by flow-cytometer (BD Bioscience, MA, USA) with the CellQuest software (BDIS).

### Mitochondrial membrane potential assessment

Mitochondrial membrane potential **(**MMP) was assessed using the Rhodamine 123 staining. 500 μl HepG2 cells (1×10^6^) were incubated at various concentrations of peiminine for 24 h. 10 μl Rhodamine 123 dye (5 g/ ml) was added to cell suspension and cells were incubated in humidified incubator at 37°C with 5% CO_2_ for 30 min. Then the cells were harvested and washed with PBS for 2 times. The fluorescence intensity and cell number were analyzed by flow cytometer (BD Bioscience, MA, USA).

### Determination of caspase activity

After treatment of HepG2 cells with peiminine alone at a concentration of 4 μg/ml or a combination of peiminine at a concentration of 4 μg/ml and z-DEVD-fmk at a concentration of 10 μmol/L, caspase-3 activities were assessed using a specific colorimetric kit (R&D Systems, Minneapolis, MA, USA) following the manufacturer’s instructions. Absorbance was read by spectrophotometer at a wavelength of 405 nm and caspase activity of caspase-3 was determined.

### Intracellular signaling array

Cell lysates were prepared as described above and total proteins were extracted. Intracellular signaling molecules were determined using a PathScan intracellular signaling array kit (Cell Signaling Technology, MA, USA) according to the manufacturer’s instructions.

### Western blotting

After treatment of HepG2 cells (1×10^**6**^) with 0, 2, 4 and 6 μg/ml of peiminine for 24h, cell lysates were prepared and centrifuged at 12,000×g for 15 min at 4°C. Next, the total protein content was extracted from the resulting supernatant and the protein concentration was quantified through the bicinchoninic acid (BCA) assay. Equal concentrations (30 μg) of protein were separated by 10% SDS-polyacrylamide gel, and transferred onto PVDF membranes. After blocking with TBST containing 5% skimmed milk for 1h, membranes were incubated overnight at 4°C with rabbit monoclonal anti-human β-actin, procaspase-3, p53, procaspase-8, procaspase-9, Bcl-2, and Bax primary antibodies, and rabbit polyclonal anti-PARP(Asp214, 89 kD), casepase-3, casepase-8, casepase-9, and cleaved PARP(Asp214, 89 kD) primary antibodies. Then, membranes were washed PBS and incubated with horseradish peroxidase (HRP)-conjugated secondary antibodies at 25°C for 1h. Proteins were visualized using an enhanced chemiluminescence (ECL) kit (Millipore, MA, USA).

### Statistical analysis

Data were presented as mean ± standard deviations (SD) from at least three independent experiments. Statistical analysis was performed by One-way ANOVA. *p* <0.05 was considered statistically significant. Software SPSS 17.0 was used for statistical analysis.

## Results

### Cytotoxicity of peiminine on cancer cells

**Fig 1:**
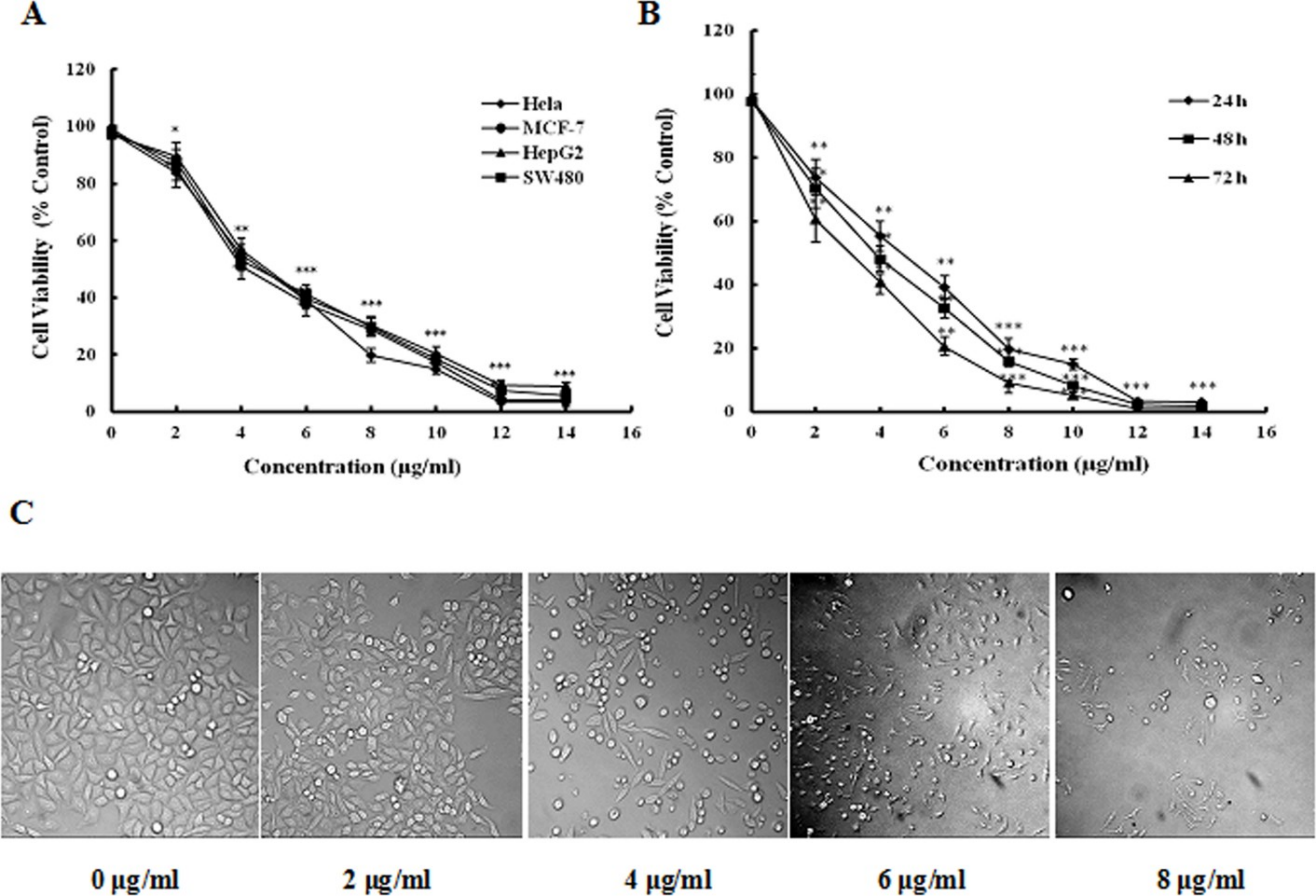
Cytotoxicity of peiminine on cancer cells. (A) The cytotoxicity of piminine upon HepG2, Hela, SW480 and MCF-7 cells was assessed by the MTT assay. (B) HepG2 cells were incubated in the presence of peiminine at indicated concentrations for 24, 48, and 72 h and cell viability was assessed by MTT assay. (C) HepG2 Cells were incubated in the presence of peiminine at indicated concentrations for 24h and cellular morphology wereobserved using an inverted microscope. Data are presented as the mean±SD from three independent experiments. *p < 0.05 vs. control (untreated cells), **p < 0.005 vs. control (untreated cells),***p < 0.001 vs. control (untreated cells).

The cytotoxicity of piminine upon HepG2, Hela, SW480 and MCF-7 cells was assessed by the MTT assay. As shown in Fig 1A, piminine exhibited a significant inhibition on the survival of HepG2, Hela, SW480 and MCF-7 cells. IC_50_ values of Hela, HepG2, SW480 and MCF-7 cell lines were 4.89, 4.58, 5.07 and 5.12 μg/ml at 24 h, respectively.

The cytotoxic effect of piminine at various concentrations during 24-, 48-, and 72 h of culture is illustrated in Fig 1B. The inhibitory effects of piminine on HepG2 cells showed concentration dependent manner and statistically significant compared to the control group. IC_50_ values of peiminine on HepG2 cells at 24-, 48-, and 72 h were 4.58 μg/ml, 4.05 μg/ml, and 3.79 μg/ml, respectively. Piminine induced integrity damage of cytoplasmic membranes, indicative of necrosis of the HepG2 cells and the population of significantly reduced (Fig 1C). The results indicated that peiminine displays the marked cytotoxicity to experimental cells.

### Peiminine-induced apoptosis of HepG2 cells

**Fig 2:**
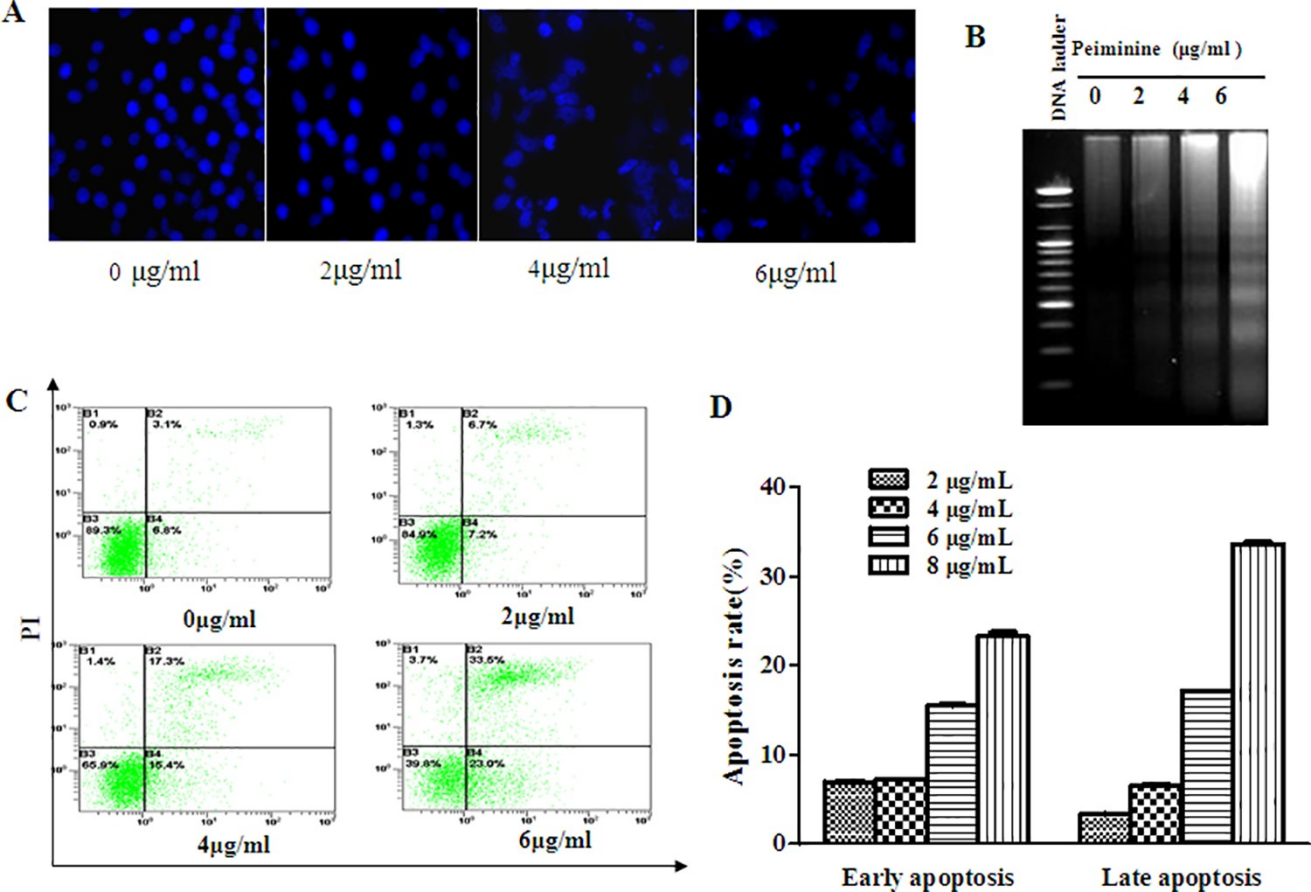
Peiminine induces apoptosis in HepG2 cells. (A) HepG2 cells treated with peiminine were stained using DAPI solution and observed by fluorescence microscopy (×400). (B) Genomic DNA of HepG2 cells treated with peiminine for 24 h was assessed by agarose gel electrophoresis. (C) Annexin V-FITC/propidium iodide (PI) staining of HepG2 cells treated with peiminine for 24 h was determined by flow cytometry. (D) The apoptotic rate of HepG2 cells exposed to peiminine for 24 h was analyzed by flow cytometry. Data are presented as the mean±SD from three independent experiments.

After treatment of HepG2 cells with peiminine at different concentrations for 24 h, apoptosis was detected by DAPI stain, DNA fragmentation and flow cytometry assay. The results showed chromatin condensation and apoptotic bodies were observed in HepG2 cells treated with peiminine (Fig 2A). The result of DNA fragmentation showed peiminine can dissociated chromosome to produce DNA fragments dose-dependently (Fig 2B). As shown in Fig 2C and Fig 2D, early apoptotic cells comprised 8.28, 10.32, 15.23, and 19.36% and late apoptotic cells comprised 3.36, 5.08, 3.55, and 5.51% after treatment of HepG2 cells with peiminine at 0, 2, 4 and 6 μg/ml, respectively.

### HepG2 cells arrest at the G2/M phase induced by peiminine

**Fig 3:**
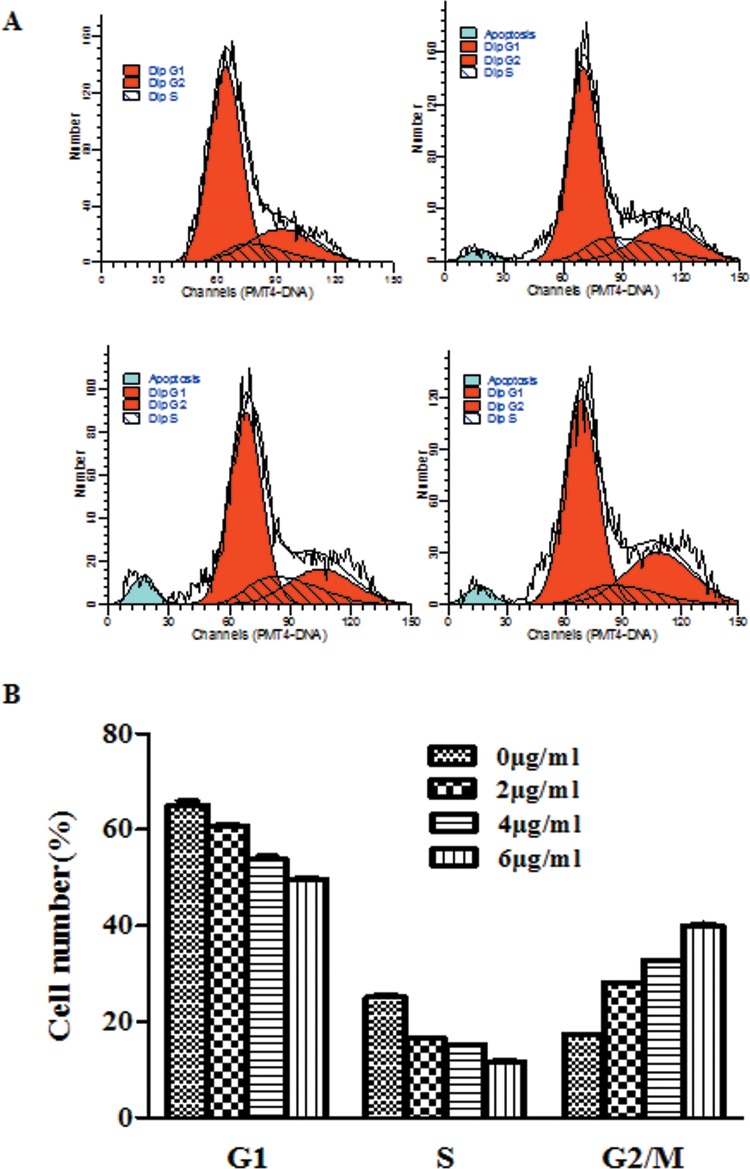
Cell cycle analysis of HepG2 cells treated with peiminine for 24 h. The values indicated the percentage of cells in the indicated phases of the cell cycle. The data shown were representative of three independent experiments.

To investigate the mechanism of the growth suppression effect induced by peiminine, cell cycles distribution of HepG2 cells treated with various concentrations of peiminine were assessed by flow cytometer. The results indicated the percentage of G1 phases of HepG2 cells treated with peiminine decreased from 65.15% ±0.78 to 49.55%±0.17 with the increase of concentrations. The percentage of S phases decreased from 25.10% ±0. 52 to 11.68%±0.16 with the increase of concentrations. Meanwhile the percentage of G2/M phases increased from 17.32% ±0.20 to 39.99%±0.47 with the increase of concentrations. (Fig 3)

### Mitochondrial membrane potential reduction induced by peiminine

**Fig 4:**
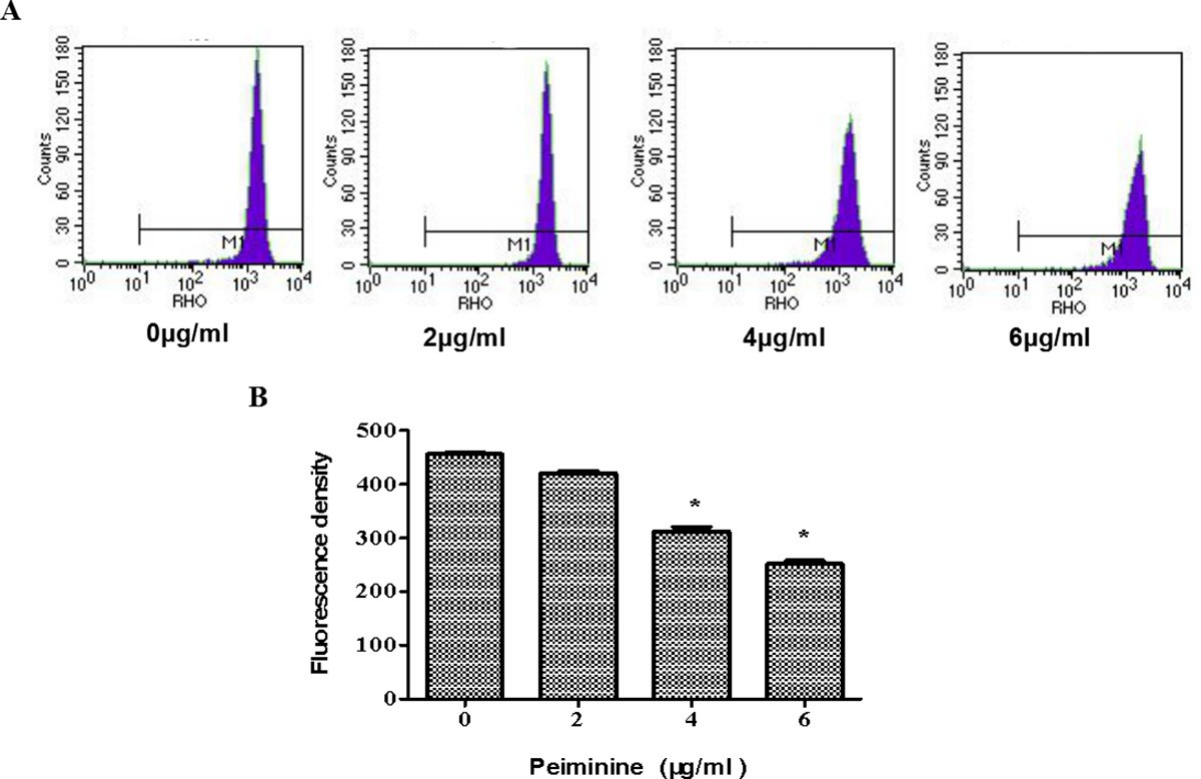
Mitochondrial membrane potential reduction induced by peiminine. After peiminine treatment at different concertrations, the mitochondrial transmembrane potential of HepG2 cells was determined by flow cytometry. Cells were stained using the Rhodamine 123 dye at 2.5 μg/ml for 15 min, and mean fluorescence intensity was determined by flow cytometery. *p < 0.05 vs. control (untreated cells).

Mitochondria activate apoptotic cell death by controlling the level of released proapoptotic proteins [12]. They also have important roles in non-apoptotic cell death [13]. In this study, the results showed that the number of living cell treated with peiminine signaficantly decreased (Fig 4A) and mitochondrial membrane potential significantly decreased in a concentration-dependent manner (Fig 4B).

### Activity of caspase-3 promoted by peiminine

**Fig 5:**
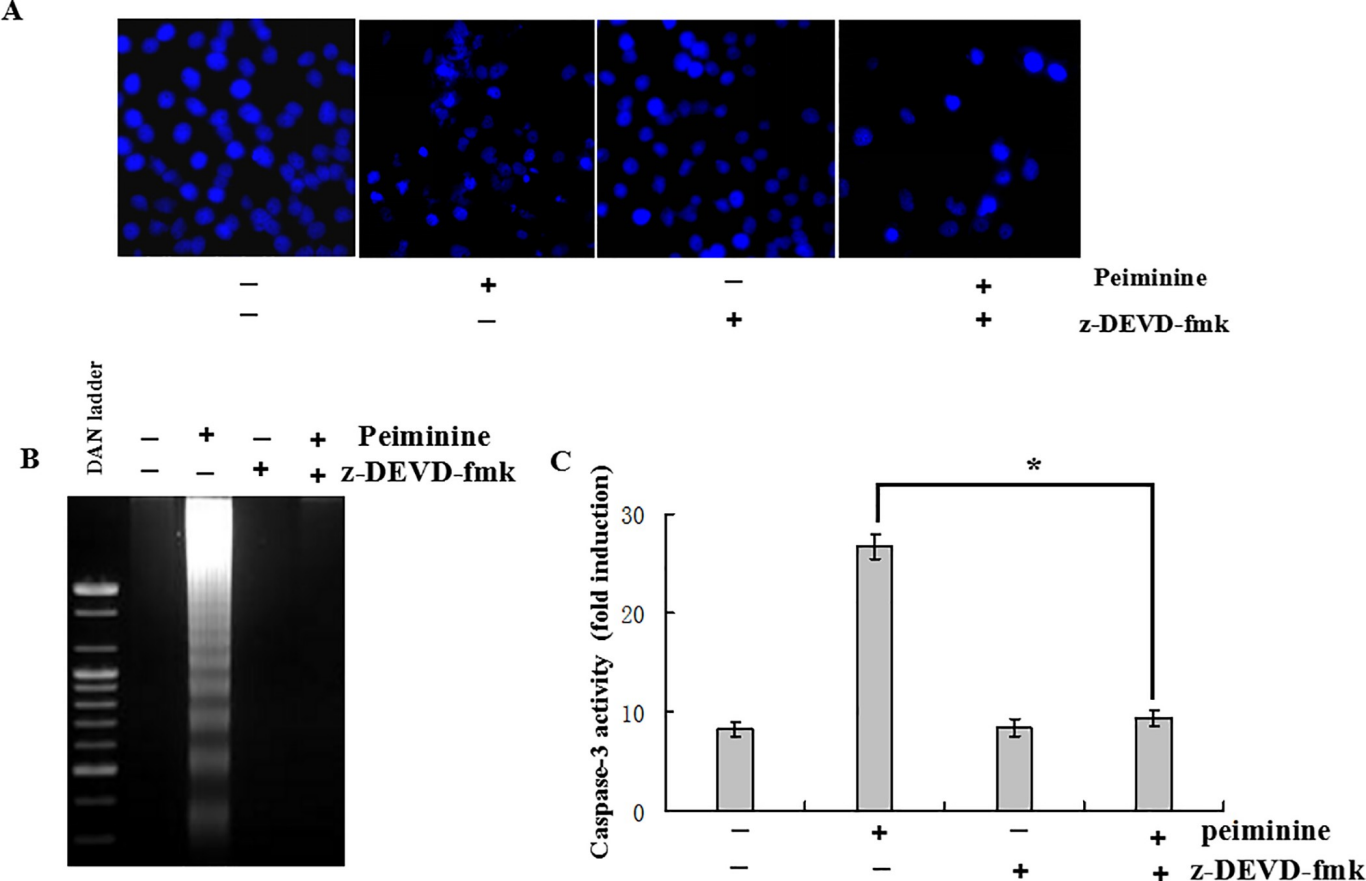
Peiminine-induced apoptosis in HepG2 cells is inhibited by caspase-3 inhibitor. (A) DAPI staining and fluorescence microscopy analysis. (B) DNA fragmentation assessment by 0.8% agarose gel electrophoresis. (C) Evaluation of caspase-3 activity using DEVD-pNA as substrate. Data are presented as the mean±SD from three independent experiments. *p < 0.05 *vs*. control (untreated cells).

z-DEVD-fmk (Z-Asp[OMe]-Glu-[OMe]Val-Asp[OMe]-CH2F) is a cell-permeable, irreversible inhibitor of Caspase-3/CPP32[14]. In this study caspase-3 inhibitor was employed to further assess the role of caspase-3 activation in peiminine-treated cells. The results showed that z-DEVD-fmk prevented chromatin condensation of peiminine-treated cells (Fig 5A). Moreover, DNA fragmentation also showed that z-DEVD-fmk prevented the occurrence of DNA fragmentation in peiminine-treated cells. Pretreatment of cells with z-DEVD-fmk (50 μM) markedly decreased caspase-3 induction (Fig 5B). The activity of caspase-3 in HepG2 cells significantly increased after treatment with peiminine. However, pretreatment of cells with z-DEVD-fmk (50 μM) markedly decreased peiminine- induced caspase-3 activity (Fig 5C).

### Signaling molecules of peiminine-induced apoptosis

**Fig 6:**
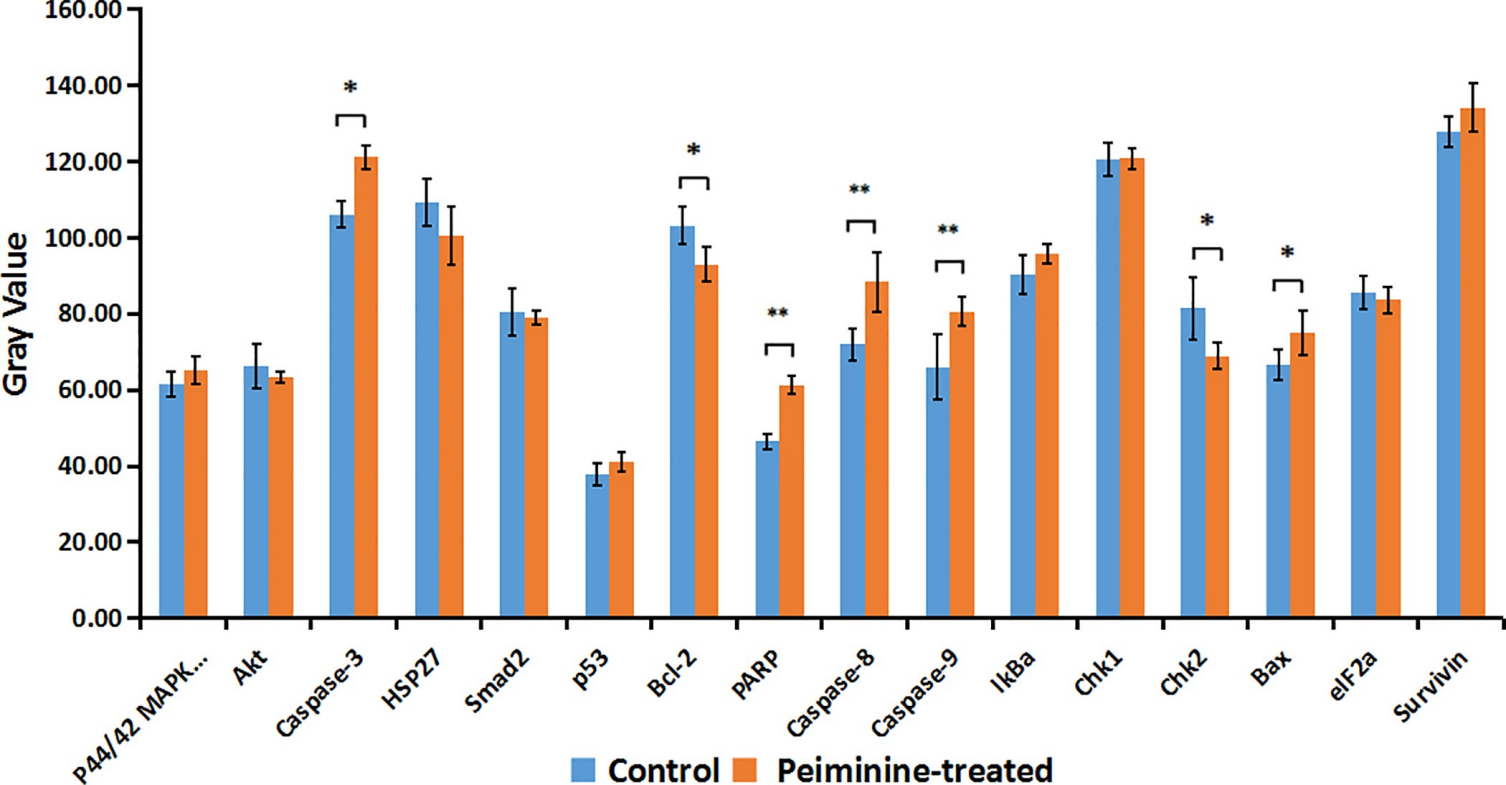
Signaling molecules of peiminine-induced apoptosis. After peiminine treatment, total proteins of HepG2 cells were extracted and intracellular signaling molecules were determined using a PathScan intracellular signaling array kit. Data are presented as the mean±SD from three independent experiments. *p < 0.05 *vs*. control (untreated cells), **p < 0.005 vs. control (untreated cells).

To further elucidate the molecular mechanisms of peiminine-induced apoptosis in HepG2 cells, a PathScan intracellular signaling array kit was used to determine the changes of signaling molecules in HepG2 cells before and after peiminine treatment. The data showed that the expression of Bax, cleaved PARP, and Caspase-3,8,9 were significantly up-regulated in peiminine-treated HepG2 cells. In addition, the expression of Bcl-2 and Chk2 were down-regulated in HepG2 cells after treatment with peiminine (Fig 6).

### Effects of peiminine on the expression of apoptotic-related proteins

**Fig 7:**
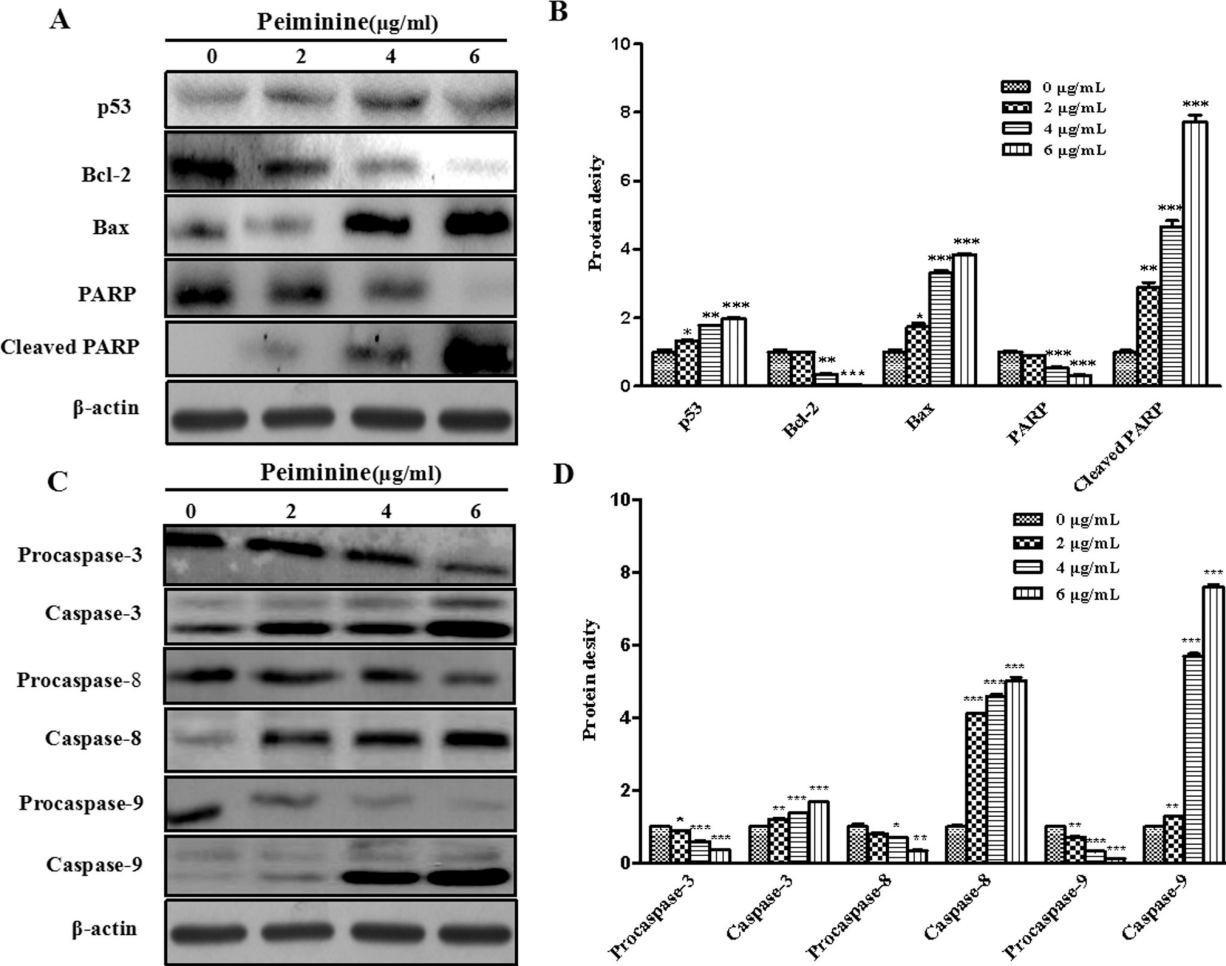
Expression of peiminine-induced proteins related to proliferation in HCC cells. (A) Expressions of p53, Bcl-2, Bax, PARP, and cleaved PARP in HepG2 cells treated with peiminine were determined by Western blot analysis. (B) Expressions of procaspase-3, -8, and -9, caspase-3, -8, and -9 in HepG2 cells treated with peiminine were determined by Western blot analysis. Data are presented as the mean±SD from three independent experiments.*p < 0.05 vs. control (untreated cells), **p < 0.005 vs. control (untreated cells),***p < 0.001 vs. control (untreated cells).

To further unveil the mechanism of peiminine-induced apoptosis, various key effectors of programmed cell death were quantitated at the protein level. Caspase-3 is essential for changes observed during apoptosis[15]. Our results showed that the expression of procaspase-3, PARP_1_(Asp214, 89 kD), procaspases-8 and -9, and Bcl-2 were significantly reduced by 2-6 μg/mL of peiminine treatment (Fig 7A and 7B). Conversely, caspase-3, 8, 9, PARP_1_ (Asp214, 89 kD) cleaved and Bax protein levels were significantly increased upon peiminine treatment (Fig 7C and 7D).

## Discussion

It is reported that peiminine can inhibit colorectal cancer cell proliferation by inducing apoptosis and autophagy and modulating key metabolic pathways[10]. The present study using the MTT assay to investigate the inhibitory effect of peiminine on proliferation upon cancer cell *in vitro*. Our findings demonstrated that peiminine could significantly inhibit the proliferation of Hela, HepG2, SW480 and MCF-7 cells and the IC_50_ values at 24 h were 4.89, 4.58, 5.07 and 5.12 μg/ml in Hela, HepG2, SW480 and MCF-7 cell lines. The further study indicated that peiminine markedly inhibited HepG2 cell viability in a time- and dose-dependent manner. For HepG2 cells, IC_50_ values of 4.58 μg/ml, 4.05 μg/ml, and 3.79 μg/ml were obtained at 24, 48, and 72 h, respectively. Additionally damage of cytoplasmic membranes and necrosis of HepG2 cells treated with peiminine were observed. These results indicate that peiminine has potent cytotoxicity toward the experimental cells and can inhibit cellular proliferation in a time- and dose-dependent manner.

Apoptosis is a fundamental mechanism of cell programmed death that can be activated by a variety of extracellular and intracellular insults. Anticancer drugs often inhibit tumor cells by inducing apoptosis [16]. Apoptotic cells are characterized by several unique features, including cell shrinkage, chromatin condensation, DNA fragmentation, cell surface expression of phosphatidylserine, and membrane blebbing [17]. In this study the morphological changes of nuclear in HepG2 cells in response to peiminine treatment were determined by fluorescence staining and DNA fragmentation assay. The results demonstrated apoptotic morphological changes including cell shrinkage, nuclear condensation and fragmented nuclei in HepG2 cells treated with peiminine. These apoptotic effects were further validated by the significant increase in the apoptotic cell population after peiminine treatment(Fig 3).

It is known that cell-cycle dysregulation is a hallmark of tumor cells. Many anti-tumor and DNA-damaging agents induce apoptosis by arresting the cell cycle at the G1, S, or G2/M phases[18]. In our study, flow cytometry was used to determine if peiminine altered the cell cycle of HepG2 cells. The results indicated the percentages of G1 and S phases of HepG2 cells treated with peiminine decreased with the increase of concentrations. Meanwhile the percentage of G2/M phases increased with the increase of concentrations.

Apoptosis contributed to cell homeostasis and several other physiological events [12,13]. Two major pathways can activate caspases and apoptosis in mammalians, including the Fas /tumor necrosis factor (TNF) death receptor (extrinsic) and mitochondrial (intrinsic) pathways[15, 19]. The extrinsic pathway involves caspase-8 activation, whereas the intrinsic pathway involves Bax, caspase-9, and caspase-3 [20]. Caspase activation results in apoptotic cell death due to death signals from cell-surface receptors, mitochondria, or the endoplasmatic reticulum (ER)[21], with caspase-3 mainly initiating apoptosis[22]. Caspase-3 activation is induced by initiator caspases, e.g. caspase-8 or -9[23].In this study we employed caspase-3 inhibitor to further assess the role of caspase-3 activation in peiminine-treated cells. The result indicated that the potent caspase-3 inhibitor z-DEVD-fmk inhibited the appearance of numerous fragmented nuclei in peiminine-treated cells, and attenuated caspase-3 activation.

Regulation of oxidative stress plays an important role in both tumor development and responses to anticancer therapies. Under normal physiological conditions, the generation of reactive oxygen species is balanced by oxidative stress response mechanisms[10]. The present study found a considerable decrease in S-adenosylhomocysteine (SAH) levels in cells treated with peiminine, which functions as a major cellular anti-oxidant [24]. Levels of several reactive oxygen species-related metabolites, including myo-inositol and taurine, increased in peiminine-treated cells[10]. Recent biological studies suggest that antioxidant rich plants induce apoptosis in many types of cancers (eg.colon, breast, liver) [25,26]. Pro-apoptotic p53 signaling and anti-apoptotic NFkB signaling play critical roles in tumor development and progression, and are involved in angiogenesis, metastasis, and cell survival [27, 28,29]. AHM et al. [30] reported the tumor-suppressive effect of the antioxidative fraction of white mulberry is likely due to apoptosis mediated by p53 and NF kB signaling. It is well recognized that whether a cell becomes committed to apoptosis partly depends upon the balance between proteins that mediate cell cycle arrest and cell death (e.g. p53, PARP-1, Bax) and proteins that promote cell viability (e.g. Bcl-2 and Bcl-xL) [31,32].

In present study, our findings suggested that the expression of p53 was increased in a dose-dependent manner in HepG2 cells and the up regulation of p53 led to subsequent binding and down regulation of Bcl-2 expression. Down regulation of Bcl-2 and PARP increases Cleaved PARP and Bax, thereby decreasing the Bcl-2/Bax ratio and promoting apoptosis and death.

Caspase activation is a feature of apoptosis induction in response to death-inducing signals originating from cell surface receptors, mitochondria, or the endoplasmic reticulum. In response to diverse stimuli, proapoptotic Bcl-2 family proteins such as Bax initiate the intrinsic apoptotic pathway by forming channels on assimilating into the mitochondria, thus increasing outer mitochondrial membrane permeability, and thereby facilitating the release of cytochrome c and other pro-apoptotic factors from the mitochondrial inter-membrane space. Released cytochrome c forms an apoptosome complex with Apaf-1, which activates caspase 9, and in turn, its downstream caspase-3, resulting in the morphological features of apoptosis[33]. In this study, the results showed expressions of procaspase-3, procaspases-8 and -9 significantly decreased in peiminine treated HepG2 cells. Conversely, caspase-3, 8, 9 protein levels markedly increased upon peiminine treatment.

## Conclusion

In this study, we found peiminine displays significant cytotoxicity toward experimental cancer cells and inhibited cellular proliferation in a time- and dose-dependent manner. The apoptotic effects were further validated by the significant increase in the apoptotic cell population and apoptotic characteristic in peiminine-treated HepG2 cells. Peiminine can down-regulate the expressions of Bcl-2, procaspase-3, procaspase-8, procaspase-9, and PARP and up- regulate the expressions of Bax, caspase-3, caspase-8, caspase-9, and cleaved PARP. These findings demonstrate that the apoptotic effect of peiminine is mediated through both extrinsic and intrinsic apoptotic pathways in HepG2 cells.
